# Retraction: Epitope-based peptide vaccine design and elucidation of novel compounds against 3C like protein of SARS-CoV-2

**DOI:** 10.1371/journal.pone.0362333

**Published:** 2026-11-09

**Authors:** The PLOS One Editors

The *PLOS One* Editors retract this article [1] because it has come to light that the article’s peer review process was compromised. There are also concerns about compliance with PLOS policy on Manipulation of the Publication Process.

SAS did not agree with the retraction. MS (first author), SM, HM, SS, and MS (corresponding author) either did not respond directly or could not be reached.
